# In-house or outsourced public services? A social and economic analysis of the impact of spending policy on the private wage share in OECD countries

**DOI:** 10.1177/0020715217726837

**Published:** 2017-08-29

**Authors:** Nicola Pensiero

**Affiliations:** University College London, UK

**Keywords:** Government outsourcing, income inequality, power relations, public sector employment, spending policy, wage share

## Abstract

This article analyses the relationship between government spending and the distribution of private income between capital and labour. While most previous research assumes that government spending redistributes in favour of the less wealthy, I distinguish between types of expenditures that enhance the bargaining position of labour – that is, unemployment benefits, public sector employment and investment in new capital – and labour-saving and pro-business types of expenditures – that is, outsourcing to private firms. The results are derived from various panel regression techniques on a panel of 19 Organisation for Economic Co-operation and Development (OECD) countries in the period 1985–2010 and show that expenditures on public sector employment and, to a lesser extent, on new capital prevented the private wage share from declining further, even after controlling for labour market institutions, globalisation and technological change. Conversely, expenditures on outsourcing substantially contributed to reducing the private wage share. Unemployment benefits had a non-significant and negative effect on the private wage share because their increase was the consequence of higher levels of unemployment rather than policy. Implications for theory and policy are drawn, including the support for a public employment-led spending policy.

## Introduction

This article contributes to the extant literature on the private wage share by proposing a political economy analysis of the effectiveness of spending policy on the distribution of income between labour and capital. Over the period 1985–2010, the distribution of output between capital and labour shifted in favour of capital in advanced countries as shown by the decline of the private wage share from 56 to 49 percent points in our sample of 19 Organisation for Economic Co-operation and Development (OECD) countries. Proponents of power relations theory link the decline of the wage share to the changes over time of the bargaining and power relations between capital and labour (Esping-Andersen, 1985; Hicks, 1999; Korpi, 2006). By contrast, the neoclassical economic theory approach to income inequality posits that the distribution of income among workers and between workers and capital reflects their relative productivity. It argues that technological change since the 1980s, by increasing the productivity of capital and skilled workers, has shifted the distribution of income in favour of capital and skilled labour. Yet, the failure of the neoclassical approach to explain the bulk of the wage share decline (Stockhammer, 2009) leaves scope for a political economy analysis of the wage share, which focuses on the progressive weakening position of labour in the political, economic and global spheres of bargaining (Kristal, 2010).

Empirical research shows that unionisation and strike activity, labour-affiliated governments, globalisation and welfare state development are associated with a larger wage share of income (Kristal, 2010; Stockhammer, 2015). However, much less is known regarding the distributional consequences of different spending programmes.

Most extant research assumes more or less explicitly that government expenditures on public services redistribute in favour of the less wealthy (Huber and Stephens, 2014; Kristal, 2010; Stockhammer, 2015), overlooking the net and potentially conflicting effects of different spending programmes on the private wage share. Conversely, this article considers that the effect of the spending policy on the distribution of power and income between capital and labour is heterogeneous. To explore this heterogeneity, I break down state expenditures into production-related programmes, consisting of spending on public employment, outsourcing to private firms and investment in new capital, and benefits to the unemployed. Building on power relations theory, I distinguish between types of expenditures that enhance the bargaining position of labour – that is, unemployment benefits, expenditures on public sector employment and investment in new capital – and labour-saving and pro-business types of expenditure – that is, outsourcing to private firms the provision of public services.^1^ The first type of expenditure is hypothesised to enhance the bargaining position of labour by creating better alternatives to low-paid jobs in the private sector and unemployment: unemployment benefits guarantee an acceptable standard of living to the unemployed, thus reducing the economic cost of unemployment; expenditure on public sector employment creates well-paid job opportunities for low- and medium-skilled employees, which compete with jobs in the private sector; the investment in new capital requires the contractors to use labour-intensive processes. Yet, government spending can also contribute to the decline of the private wage share by outsourcing production processes to private firms, which tend to adopt labour-saving production processes.

This article analyses the impact of those mechanisms on the distribution of private income between labour’s compensation and capital’s profits. As capital income is highly concentrated and only a minority of workers obtain earnings from dividends, deposits and houses, the distinction between firms which generate capital income and wage-earning workers can be regarded as a good approximation of how income is distributed (Kristal, 2010, 2013a). The use of the private sector measure of income obviates the problem of treating the spending on public sector employees both as an explanatory factor and as (part of) the outcome.

The assembled dataset includes rich data on labour market institutions, globalisation and technological change apart from spending programmes. The impact of spending programmes is assessed on a panel of 19 countries for the period 1985–2010 using various panel regression techniques.

Based on my analysis, I argue that the increasing prevalence of government outsourcing reduces the ability of the public sector to reduce income inequality.

## Can the spending policy influence the private wage share?

### Spending policy and power relations

This article is premised on the argument that markets are embedded in social and political institutions and governments have a wide range of tools to affect the operation of markets and the creation and redistribution of income, including spending programmes (Hacker and Pierson, 2010; Jacobs and Dixon, 2006; Kenworthy, 2010). Sociological and political science research regards government spending policies as the result of political contention, where working-class affiliated parties tend to foster the well-being of their constituency through redistributive policies, while pro-business parties tend to ensure the accumulation of profits. In the aftermath of World War II, the social democratic model, in which leftist political parties were either government parties or major opposition parties, contributed to increase the wage share through pro-labour policies. In the 1980s and 1990s, labour parties lost power in many countries or were unable to support pro-labour policies as they did before (Esping-Andersen, 1985; Hicks, 1999).

The expansion of the public sector was a key component of the economic policy after World War II. Particularly relevant to this analysis is the fact that public sector employment traditionally provided opportunities for generously paid jobs for medium- and low-skilled individuals. With the explicit aim of reducing gender inequality and achieving full employment, public sector services such as health, education and social services, absorbed women, disadvantaged and low- and medium-skilled groups, which in the private sector would have earned less and would have had worse employment conditions (Esping-Andersen, 1990; Gornick and Jacobs, 1998; Lucifora and Meurs, 2006). The notion that medium- and low-skilled occupations are better paid in the public sector is also confirmed by the data provided by the European Union (EU) KLEMS database, where compensation figures are broken down by skills categories and sectors. Over the period 1985–2004, data confirm, in general, that in 12 OECD countries medium- and low-skilled occupations in the public sector are better paid than comparable occupations in the private sector, respectively 18 and 12 percent higher.^2^ This is a known fact in sociological and political economy research, yet an in-depth analysis of the distributional consequences of the relative advantage of public sector occupations is missing.

The between-sector pay gap for medium- and low-skilled occupations implies that larger expenditure on government employment increases the opportunities of relatively well-paid and better-protected jobs which workers can resort to as an alternative ‘outside’ option to the jobs in the private sector. The likely distributional effect of the public sector job opportunities is similar yet opposite to that of globalisation. Like imports from countries where labour is less costly, which places workers in developed countries in competition with lower paid workers, the availability of highly paid and more secure jobs in the public sector places jobs offered in the private sector in competition with better-paid jobs in the public sector. As the government is a wage setter and is not exposed to market competition, but only to political pressures (Pontusson et al., 2002), the likely result of this competition is a stronger leverage of workers in the private sector to bargain a larger share of income. Therefore, the competition between the government and firms pushes upwards the wages for middle- and low-skilled occupations in the private sector.

The generosity and coverage of unemployment benefits are also expected to strengthen the bargaining position of labour by reducing the cost of unemployment. Esping-Andersen (1990) argued that the range of options and the extent of benefits offered to workers in generous welfare state systems are such that they undermine the logic of pure exchange in labour markets and constrain the employer’s treatment of labour as a commodity. In other words, generous unemployment benefits decommodify workers and undermine the efficacy of the threat of unemployment as a bargaining strategy. This implies that unemployment benefits exert an upward pressure at the bottom of the distribution of private wages.

On the basis of the above discussion, I hypothesise that larger expenditures on government employment and unemployment benefits are associated with a larger wage share of private income.

A major trend of the spending policy from the 1980s is the outsourcing of production services previously carried out by public sector enterprises to private firms. Outsourcing is defined here according to the internationally agreed System of National Accounts (SNA) as goods and services provided by private companies, excluding capital, and used as intermediate inputs by the government in producing public goods and services.^3^

Over the past decades, the involvement of private firms in the provision of public services, through the government’s outsourcing of part of the production of public goods/services and the government’s acquisition of new capital, has grown. In 1985, the governments in our sample of OECD countries spent 3.3 percent of the gross domestic product (GDP) to outsource intermediate processes to private firms, slightly less than half (47%) of the expenditures on the in-house production through public sector employment. The expenditure on outsourcing increased at a yearly rate of 4.3 percent and reached 6.9 percent of the GDP in 2010, which corresponds to 65% of the expenditures on public sector employment. The expenditure on capital formation increased from 2 to 3.4 percent of the GDP over the period of interest (Table 1). These trends imply that the world of firms received larger flows of funds from the government, while the relative flow received by labour through expenditure on public sector employment decreased.

**Table 1.** Levels in selected years and yearly percent change of selected government spending programmes in 19 OECD countries.

	1985 level	Percent average change per year	2010 level
Outsourcing % GDP	3.3	4.3	6.9
Government outsourcing/government wages	0.47	1.5	0.65
Government capital formation % GDP	2	2.8	3.4

OECD: Organisation for Economic Co-operation and Development.

Countries: Austria, Belgium, Canada, Denmark, Finland, France, Germany, Greece, Ireland, Italy, Japan, the Netherlands, Norway, Portugal, Spain, Sweden, Switzerland, the United Kingdom and the United States.

Gross domestic product (GDP) is the potential measure of GDP.

The relative increase in outsourcing was part of a broader push towards privatisation, which was proposed as a reaction to the problematic economic viability of some State-owned Enterprises (SOE) (Megginson and Netter, 2001). To address the losses and accumulated amounts of debt that some SOE encountered, many states undertook not only financing measures such as bailing out but also restructuring and privatisation. In general, the goals proposed by the proponents of privatisation programmes are to reduce government interference in the economy, raise revenue for the state and promote economic efficiency via market discipline, with equity issues such as equal access and redistribution not being primary objectives (Hermann, 2015). The spread of outsourcing measures across developed nations shows that these goals were accepted and pursued from across the partisan spectrum represented in national governments, although with varying speeds.

The impact of privatisation measures on employment, employment conditions and employment relations tends to worsen distribution. In preparing for privatisation and in the post-privatisation period, employment in the privatised enterprises declines as a cost-cutting measure and the workers who are kept are subject to work intensification and fragmentation of work conditions and wages (Birdsall and Nellis, 2003). In the newly privatised enterprises, non-managerial workers experience wage cuts as showed by the case of the privatised German hospitals (Schulten and Bohlke, 2012). While the wages of doctors show no cross-sector difference, non-medical staff and assistant nurses are paid less than their counterparts in the public sector. Overall, privatisation can imply that the same work is carried out with ‘fewer people in a non-union setting at lower levels of wages and benefits’ (International Labour Organization (ILO), 1999: 23, cited in Bayliss, 2002: 617). Therefore, I hypothesise expenditures on outsourcing to be associated with a lower wage share of private income.

Government investments in capital formation comprise expenditures on transport infrastructure, office buildings, housing, schools, hospitals and expenditures in research and development. Investments in capital generally embody labour-intensive processes and sophisticated technologies, such as research and investments in space procurement and highway construction, and therefore can positively affect the private wage share.

### Power relations in the economic sphere

While recent studies on bargaining power focused on the role of globalisation in strengthening the position of capital, traditionally, bargaining power was conceptualised as labour market institutions, such as the centralisation of bargaining settings, employment protection legislation and union activity. In centralised bargaining settings, the scope of the bargaining is to fix wages at the national level, and workers tend to promote their interests collectively. As the negotiations are for the benefits of several employees, unions and workers have an interest in negotiating in a coordinated way. The organisational unity deriving from a centralised bargaining setting renders the employees’ struggle and strike activity more effective at extracting a larger share of the output (Kristal, 2013a).

Empirical studies show that union activity and the centralisation of the wage coordination account for part of the trend of wage growth and income inequality (Bengtsson, 2014; Calderón and Chong, 2009; Kenworthy and Pontusson, 2005; Kollmeyer, 2017; Kristal, 2010, 2013a). Unionisation rates were high in the decades after World War II (between one-third and two-thirds of all workers in advanced countries were members of a union (Western, 1997)), which led unions to successfully demand higher wages and better working conditions. Unions lost members in advanced countries from the 1980s, which meant that the effect of union activity (such as strikes) on pushing upwards employees’ wages weakened (Rosenfeld, 2006). However, the loss of members does not always translate into less union activity and therefore into a loss of power for unions, at least in European countries. Unlike the United States, in European countries wage agreements are often extended, through legal or voluntary processes, to non-union members and additional sectors and firms. For this reason, union coverage (defined as employees covered by wage bargaining agreements as a percentage of all employees) declined at a slower pace than union density over time, leading some scholars to question the validity of union density as an indicator of union bargaining power and to prefer the centralisation of bargaining settings and union coverage (Fiori et al., 2012; Wallerstein et al., 1997).

The study by Kristal (2010) is particularly relevant as it uses, similarly to this study, the wage share as the measure of inequality. Apart from a significant positive effect of non-military government spending and a negative effect of globalisation, this study also found a positive effect of unionisation and strike activity on the wage share. I will include unemployment protection legislation, union density and centralisation of bargaining as control factors of the bargaining power in the economic sphere.

### Technological change and globalisation

A large part of the recent literature on income inequality analyses the impact of globalisation and technological change. Technological change is the cornerstone of the neoclassical economic theory of income distribution. The argument assumes perfectly competitive markets where incomes are determined by marginal productivity. In its modern version, it posits that since the early 1980s, technological change – and in particular information and communication technology – has been complementary to skilled labour and a substitute for unskilled labour. This implies that technological change increases the demand for skilled labour and decreases it for low-skilled workers (Autor et al., 1998; Card and DiNardo, 2002). Two contrasting mechanisms arise: the wage share of the highly skilled and more productive workers increases, while the wage share of the low-skilled and less productive workers decreases. If the latter mechanism prevails, then the wage share overall is penalised. Another characteristic of recent technological change that could lead to a lower wage share is the capital augmenting effect. In this case, technology increases capital productivity more than labour productivity, which leads firms to shift the demand away from labour and in favour of capital, thereby decreasing the wage share (European Commission (EC), 2007; International Monetary Fund (IMF), 2007).

The evidence in support of the technological change hypothesis in relation to developed countries is mixed. From a neoclassical perspective, EC (2007) and IMF (2007) found that technological change is the single most important determinant of the decline in the wage share, while Stockhammer (2009) found a modest effect for it. Kristal (2013b) focused on the wage share within US industries and found support for the hypothesis that technological change negatively and significantly affected the wage share in the United States indirectly through skill polarisation and anti-union strategies.

The traditional globalisation hypothesis is that in a system in which capital and labour are diversely distributed in rich and poorer countries, the abundant factor will gain in each system type. In rich countries, capital is more abundant, whereas labour is abundant in developing countries. Globalisation is thus expected to benefit capital in rich countries and labour in less rich countries. It exerts a downward pressure on the wages of unskilled workers in rich countries while increasing income from capital, raising inequality within these economies (Kanbur, 2000). This line of reasoning assumes, however, that both capital and labour are not mobile, which is unrealistic given the recent marked increase in capital mobility. Another issue to consider is that labour is not homogeneous: although wages decline and jobs are lost in some sectors in rich countries, other sectors may benefit from international trade (EC, 2007; Richardson, 1995; Stockhammer, 2009).

The political economy interpretation of globalisation is that it contributes to an increase in economic inequality by erasing the spatial barriers that protect domestic labour from multinational competition and weakening the influence of domestic political forces on the labour market. Globalisation places domestic workers in competition with workers from other countries, where domestic firms have the option of relocating production, importing or exporting goods. Empirical research shows a high degree of consensus that globalisation is detrimental for the wage share not only in developed countries, as the traditional globalisation hypothesis would predict, but also in developing countries (Stockhammer, 2015). Its impact on income inequality is less clear, probably owing to the variability of its impact depending on other moderating factors. Some studies find a negative effect on income inequality in low- and middle-income countries, while others find significant detrimental effects in developed countries as well (Bergh and Nilsson, 2010) and within developed countries, particularly on the wages of unskilled workers (Wood, 1995). Kollmeyer (2015) analysed the role of government spending as a moderator of the impact of globalisation and found that globalisation increases inequality only in countries with relatively small public sectors, while for countries with larger public sectors globalisation can occur without leading to a more unequal distribution of income.

Finally, the level of industrial employment can affect income inequality. Its decline in all advanced post-industrial economies, although to different degrees, correlates with the decline in the wage share. The industrial sector – it is traditionally posited – tends to offer better-paid jobs for low-skilled workers than the service sector does, hence its potential relevance for income distribution. The effectiveness of the level of industrial employment on inequality depends on the pattern of industrialisation though. A prevalence of industries which employ a higher proportion of low-skilled workers and use domestic raw materials and labour-intensive technology can have equalising effects on the distribution of income between capital and labour. By contrast, when the prevailing production process within industries is capital-intensive and requires fewer highly paid and highly skilled workers, the effect on the distribution between wages and profits is expected to increase inequality.

## Analysis

### Data

The panel I use consists of 19 OECD countries over the period 1985–2010.^4^ The countries are Austria, Belgium, Canada, Denmark, Finland, France, Germany, Greece, Ireland, Italy, Japan, the Netherlands, Norway, Portugal, Spain, Sweden, Switzerland, the United Kingdom and the United States.

The variables used are from diverse sources created with more specific goals. The dependent variable is the private wage share of GDP (functional income distribution).

The source of the adjusted private wage share of GDP is the Annual Macro-Economic Database (AMECO, EC). In order to ovoid endogeneity in analysing the effect of public sector employment, I analyse the effect of the public sector employment on the private wage share. Indeed, the public employment is part of the overall employed population, which renders the relationship between public sector employment and the total wage share an accounting identity. The adjustment consists in imputing wage earnings for the self-employed and aims to avoid counting all earnings of the self-employed as profits. It is routinely adopted in the literature and directly included in the AMECO dataset. In defining the private wage share, I follow Stockhammer (2015). The wage share of the total economy (W) is the sum of the private wage share ($W^{p}$) weighted by its size and the government wage share ($W^{g}$). The measure of the size of the private sector wage share is $1-W^{g}$. Therefore

$$W=\left(1-W^{g}\right)*W^{p}+W^{g}$$

It follows that the private wage share is given by

$$W^{p}=\frac{W-W^{g}}{1-W^{g}}$$

The weighted private wage is relative to the private sector only and thus avoids that the measure of the private wage share is influenced by the size of the government sector.

I distinguish between government expenditures allocated to the production of public goods and services and unemployment benefits.^5^ Production expenditures, in turn, consist of wages of employees in government, capital formation and costs to contract out or outsource to firms in the private sector part of the production processes of public goods and services, excluding capital, which are provided by the government.

The government investment in public employment is captured by the overall compensation of employees or *government wages* and is from the OECD National Accounts at a Glance database. The measure is relative to the potential GDP, which is a hypothetical aggregate output that would be realised if the economy was operating at potential or full employment. The potential measure of GDP, by removing the effect of fluctuation of GDP due to the economic cycle, better captures the policy action. By contrast, the use of an unadjusted measure of the GDP as the denominator conflates the effect of policy and economic cycle. For example, during an economic recession, the decline in GDP (denominator) would push up the index of government wages, regardless of the variation in expenditures on government wages. Therefore, the adjusted measure of the GDP better reflects policy and provides a more valid basis for drawing policy implications. Similarly, the use of the potential GDP in constructing the index of trade openness removes the fluctuations due to economic cycles and captures the structural openness of the economy. The potential measure of the GDP is from the OECD Economic Outlook database.

The second component of production-related expenditures is expenditures on outsourcing state functions (*government outsourcing*):

While compensation costs are an important part of the production costs of goods and services in the public domain, governments also spend a large amount of resources on outsourcing to buy goods and services from the private sector that are used in the short term in the production of services to government. (Pilichowski and Turkisch, 2008: 13)

The extent to which governments relies on outsourcing experienced substantial increases over time. It is plausible that some of those changes are dictated by an increasingly complex and specialised technology used in some of the services and goods provided by the government, which can be more easily provided by private firms with expertise in the sector. However, the extent of cross-country variation, both in levels and patterns of change in outsourcing, indicates that most of the prevalence of outsourcing is accounted for by policy choices. The measure is from the OECD Economic Outlook database (called intermediate consumption) and is relative to the potential GDP.

The third component of the production-related expenditures is the investments in capital formation in the government sector (*government capital formation*). This measure is expenditures on infrastructure and research and development from the OECD National Accounts at a Glance database and is relative to the potential GDP. The definition of capital formation focuses on the acquisition of new assets and does not take into consideration the depreciation of existing assets.

To capture the effect of the welfare state in reducing the cost of unemployment, I use the sum of cash and in-kind unemployment benefits relative to the potential GDP or simply *unemployment benefits*. The data are from the Comparative Welfare State dataset (Brady et al., 2014), which in turn are from the OECD Social Expenditure database, which provides comparable statistics on social spending at the programme level.

The *unemployment rate* is the number of unemployed persons as a percentage of the civilian labour force and is from the Comparative Welfare State dataset (Brady et al., 2014).

*GDP per capita* in international dollars is the chosen indicator of economic development. The logarithm of GDP per capita and its quadratic term are used to capture the non-linear, such as Kuznets type, effects of economic development on inequality (Ahluwalia, 1976). The source is the Comparative Welfare State dataset (Brady et al., 2014).

*Industrial employment* is measured as total industrial employment percent of the working age population (aged 15–64) from Comparative Welfare State dataset (Brady et al., 2014).

To capture the power relations in the economic sphere, I use three indicators: union density, wage setting coordination and employment protection legislation. *Union density* is defined as net union membership as a percentage of employed wage and salary earners and is from the Comparative Welfare State dataset (Brady et al., 2014);^6^ *wage setting coordination* captures the degree to which wage bargaining is centralised (1 = fragmented wage bargaining, 5 = economy-wide bargaining) and is from the Comparative Welfare State dataset too. The index of *employment protection* legislation is the synthesis OECD indicator of the strictness of regulation on dismissals for regular contracts. It ranges from 0 (least strictness) to 5 (most strictness). The source is the OECD Employment and Labour Force Statistics database.

Globalisation is defined empirically using two indicators: *trade openness*, which is the most common indicator of globalisation in the literature, and foreign direct investments (*FDI*). Trade openness consists of imports plus exports relative to the potential GDP and is taken from the Comparative Welfare State dataset (Brady et al., 2014). FDI captures the domestic capital mobility and is measured by the outward direct investment made by the firm of the reporting economy to external economies as a share of the potential GDP. The source is the IMF International Financial Statistics database.

The skill-biased technical change is empirically defined using the share of information and computer services, which consist of computers, software and communications equipment, divided by the gross value added (information and computer services (ICT)), which is taken from the EU KLEMS database. ICT and its squared transformation are used to evaluate the extent to which companies need a certain minimal level of technological innovation to shift the demand away from low-skilled labour and in favour of capital as hypothesised by the technological change hypothesis.

### Panel regression

I analyse the evolution of private wage share using panel regression, which offers the advantage of controlling for unobserved and time-constant characteristics using country fixed effects (FE) (Wooldridge, 2002). When dealing with panel data, the standard regression assumption of independent, identically distributed errors is likely to be violated by three potential problems: first, panel heteroscedasticity, which means that the error variances differ among countries; second, contemporaneous correlation of the errors, owing to the mutual relations among the economies in this panel. Indeed, it is possible that a shock that impacts one economy is likely to have an impact on its partner economies. Third, there is the possibility of serially correlated errors, which implies that the current private wage share is influenced by the private wage share of the past.

This latter problem requires particular caution in this analysis since my panel, like most macro panel datasets, consists of a relatively small number of years (*T*) and countries (*N*). To deal with autocorrelation, I will use two different approaches, Prais–Winsten estimator, which is a version of feasible generalised least squares (FGLS) regression, and Arellano–Bond (AB) estimator.

The Prais–Winsten regression is an FGLS estimator that (quasi) differences the error term in order to purge autocorrelation and is compatible with standard errors which correct for heteroscedasticity and contemporaneous correlation (panel-corrected standard errors (PCSEs)). The Prais–Winsten regression consists in the following steps. First, it uses ordinary least squares (OLS) regression to estimate the effect of the independent variables. It then uses the residuals of this model to fit a first-order autoregressive (AR(1)) process, where the residuals depends on the residuals of the previous year. It then applies a quasi-differencing transformation (Prais–Winsten transformation) to remove serial correlation and applies OLS to the transformed model (Judge et al., 1985).

I also use the AB estimator (Arellano and Bond, 1991) which is explicitly designed to take into account the autocorrelation of the dependent variable. Like any dynamic model, the AB estimator includes the lagged value of the private wage share as an explanatory variable to correct for the dependency of the dependent variable on its past values. The problem with this strategy is that it generates an endogeneity bias arising from the inclusion of the private wage share in both sides of the equation.^7^ The AB estimator (called first-differenced generalised methods of moments, 1991) obviates this potential bias using instrumental variables. First, it transforms the model into first differences to remove country effects. Once the FE are removed, the lagged values of the dependent and the independent variables can be used as instruments even if they are not strictly exogenous, that is, even if they are correlated with the past and current errors. The model consists of a set of year-specific equations with varying instruments, where equations of later years use additional lagged values of the instruments.^8^ On one hand, the econometric literature highlights that the use of too many instruments can result in the overfitting of the endogenous variables worsening the performance of the AB estimator. On the other hand, there is no precise rule on what is the correct number of instruments. Roodman (2009) recommends testing the results for sensitivity to the reduction in the number of instruments. I tested the results for sensitivity to the exclusions of five blocks of instruments – the indicators of spending programmes, the indicators of labour market regulation, the indicators of globalisation, the indicators of GDP per capita and level of industrial employment, and the unemployment rate. The results are sensitive to the exclusion of the globalisation and spending programmes variables; those variables, when included as instruments, tend to reduce the effect size of the explanatory variables, while the remaining instruments do not alter the effect estimates of the explanatory variables. Therefore, I decided to include only the spending programmes and globalisation indicators as instruments, apart from the private wage share.

I will combine the Prais–Winsten estimator with FE (Prais–Winsten FE, first modelling strategy) and first differencing (Prais–Winsten FD, second modelling strategy) and the AB estimator with FE (third modelling strategy). The inclusion of FE obviates the potential bias arising from the omission of unmeasured time-invariant institutional characteristics. First, differencing the equation removes country-specific time-constant effects (similar to the FE), but is preferable when there is autocorrelation.^9^

When panels consist of a relatively small number of years and countries, it is difficult to prefer one model over the other. The widely used AB estimator (Arellano and Bond, 1991) is designed for panels with a small number of years and with a relatively large number of countries, while my panel consists of a relatively small number of countries. Therefore, I decided to err on the side of caution and use an inductive approach in evaluating the results; when the effect estimates regarding a particular spending programme are significant across the three modelling strategies, I shall regard the results as a strong confirmation of the programme’s effectiveness; when the estimates are significant in two of the three modelling strategies, I shall regard the results as a substantial confirmation of the programme’s effectiveness; when the effect estimate is significant in one modelling strategy only, I shall regard the results as a weak confirmation of the programmes’ effectiveness.

Panel unit root tests that allow for the possibility that some countries’ private wage share contains unit roots while other countries’ private wage share do not, reject the hypothesis of a common unit root of private wage share at the 1 percent level.

### Determinants of the private wage share

The results of the effectiveness of spending programmes are presented in four specifications, successively adding variables pertaining to labour market institutions, globalisation and technological change. Throughout all specifications, the GDP per capita, unemployment rate and the level of industrial employment are used as controls. In choosing the variables to include in the panel regression models, I will prefer parsimony when the exclusion of a variable does not imply a loss of information. This criterion applies to two variables, union density and foreign direct investment, whose effect is not significant in any of the modelling strategies and whose exclusion does not change the effect estimate of the spending programmes. I will propose an explanation of the lack of contribution to the models of those variables when discussing the results.

The first three specifications share the same panel, while the last specification uses a reduced panel due to the availability of the technological change variable just for 14 countries and only until 2007. The 14 countries are Austria, Belgium, Denmark, Finland, France, Germany, Ireland, Italy, Japan, the Netherlands, Spain, Sweden, the United Kingdom and the United States. I preferred to use a larger panel for three of the four specifications rather than using consistently smaller samples across all four specifications.

Model I confirms the expectation regarding the positive effect of larger expenditure on government wages on the wage share of private income. The consistency of this result across the three models (Prais–Winsten FE, Prais–Winsten FD and AB) confirms that the results are robust to the different estimation strategies. In the Prais–Winsten FE model, the effect size is larger than in the AB model (0.74 vs. 0.41), but smaller than in the Prais–Winsten FD model (0.74 vs 1.39).

The three models consistently support the hypothesis of a negative effect of spending on outsourcing for the private wage share. The effect estimate of outsourcing is −0.42 in the Prais–Winsten FE model, −0.62 in the Prais–Winsten FD model and −0.15 in the AB model. The more complete specifications that I will present later confirm substantially those results. These findings indicate that the government contractors tend to use a lower proportion of labour units compared to capital and/or succeed in negotiating lower pay for their employees while directing a larger share of the government funds to profits. This is in line with a pro-business view of privatisation in which the government contractors attain profits by reducing labour units and wages.

Expenditures on capital formation are significantly and positively associated with a larger private wage share only in the Prais–Winsten FE model in this specification (0.34), although they become significant in both the Prais–Winsten FE and FD models in specifications III and IV, which will be discussed later. These findings provide substantial support for the hypothesis that the investment of governments in capital formation requires labour-intensive processes.

The extent of unemployment benefits is not significantly related to the private wage share in this and other specifications except for two models (specification IV, models Prais–Winsten FE (−0.92) and AB (−0.42)), and the direction of the effect across all specifications is, contrary to expectations, negative. One possible explanation of the negative and non-significant effect is that the variations of unemployment benefits reflected at least partly the trends in the unemployment rates rather than changes in policy. This explanation is supported by the positive correlation between the unemployment rate and the unemployment benefits in our panel (Pearson correlation coefficient is 0.40, *p* < 0.001) and is in line with Huber and Stephens (2014) which find that the generosity and coverage of social spending did not change over the last four decades and the variations over time in social spending reflect the variations in the population’s needs rather than policy action. Another possible explanation is that the unemployment benefit claims anticipate to some extent the effect of a decline in households’ earned income. When households expect their earnings to shrink in the future, they are more likely to claim unemployment benefits than in situations when unemployment is a transitory accident and future earnings are likely to remain stable. The anticipation effect in combination with the positive relationship between unemployment benefit variations and unemployment rate trends might explain the lack of a statistically significant effect estimate and its negative sign.

Moving to the controls, the unemployment rate, as expected, exerts a downward pressure on the private wage share as shown by the effect estimates in the Prais–Winsten FE (−0.37), FD (−0.31) and AB (−0.24) models.

The logarithm of GDP per capita shows a negative effect on the private wage share and its quadratic term a positive effect, which implies – in line with the Kuznets curve hypothesis – that the private wage share first declines with higher values of per capita income, and then, after reaching its minimum, rises for very high levels of per capita income. However, the effect estimate of those variables is not significant at the conventional levels in all models, with the exception of the logarithm of GDP per capita in the Prais–Winsten FE model in specification I and in all models in specification IV. The results, therefore, do not provide sufficient support for Kuznets curve hypothesis. The effect of the level of industrial employment is significant in the Prais–Winsten FE and the AB models in specifications I and II and in the AB model in specifications III and IV, confirming substantially that economic development, as proxied by this variable, followed a path in which capital disproportionally benefited from productivity gains.

In model II, I include the factors that affect the bargaining power of labour – union density, centralisation of wage bargaining and employment protection legislation. In general, those factors do not show a strong statistical effect on the private wage share. The effect of union density is not statistically significant in any of the model specifications and the estimates of the effectiveness of the spending programmes were not different from the ones obtained excluding those variables. Therefore, I decided to exclude union density from the presented analysis. Employment protection is significantly and positively associated with the private wage share using the Prais–Winsten FE model in specifications II (2.8) and III (2.4), while wage coordination only in the Prais–Winsten FE model in specification IV (0.26). Those results provide weak, if any, support for the hypothesis that labour market structures affected positively the private wage share over the period of interest. In case of employment protection legislation and centralisation of wage bargaining, the reason could be that those factors did not significantly change over the period of analysis in this panel of countries. On the other hand, union density declined over the last decades, yet a relative large proportion of workers are still covered by some form of collective wage agreement in European countries since in these countries, unlike the United States, collective agreements are extended to non-union members and additional sectors and firms. This implies that union density might not be able to capture accurately the bargaining power of unions in this panel. The estimates of the effect of spending programmes from model II remain very similar to the ones obtained in previous models, showing that spending programmes have an independent impact on the private wage share.

Specification III shows that trade openness is negatively and significantly associated with the private wage share. The effect of trade openness is significant across the three models and its size is −0.06 in Prais–Winsten FE, −0.08 in Prais–Winsten FD and −0.02 in AB models. The size and significance of the estimates of the effect of expenditures on government wages, outsourcing and unemployment benefits are similar to the previous ones, while the effect of expenditure on capital formation is significant not only in the Prais–Winsten FE model (0.42) as before but also in the Prais–Winsten FD model (0.36). A possible explanation of the more robust effectiveness of expenditures on capital formation in this specification is that the investments in labour-intensive productive processes that this type of expenditures implies are critical especially in relation to the increased competition induced by economic globalisation.

The effect of foreign direct investment is not statistically significant in any of the models and the estimates of the effectiveness of the spending programmes were not different from those obtained excluding those variables. Therefore, I decided to exclude this variable from the presented analysis. The findings of the effectiveness of the two indicators of globalisation imply that globalisation in this panel tends to affect the bargaining power of labour by inducing competition in the home labour market, rather than by providing firms with the option of relocating the production as a bargaining device.

Finally, I add the ICT variable as a control (specification IV). The number of years and countries is dictated by the EU KLEMS dataset, which is the source of the ICT variable. The effectiveness of the spending programmes is comparable to the one of the previous specification III. In the Prais–Winsten FD and dynamic models, it is not possible to reject the presumption that the technological change variable is not significantly associated with the private wage share. In the Prais–Winsten FE model, the technological change variable possibly exerts a non-linear effect on the private wage share: the linear effect is positive and non-significant (3.81) and the quadratic effect is negative (−0.30). The variation of the marginal effects for different values of the technological change variable indicates that investments in ICT of up to 5 percent of the gross value added do not affect the private wage share – after this turning point, the effect becomes negative. The results regarding the variation of the marginal effects can be obtained on request.

Overall, the analysis strongly confirms that expenditures on public-service employment and outsourcing have a significant yet opposite effect on the private wage share: the expenditures on government wages contribute positively, while the expenditures on outsourcing contribute negatively to the private wage share. Results also confirm that expenditures on capital formation have a positive effect on the private wage share, although less strongly. The results do not support the hypothesis that expenditures on unemployment benefits have a positive impact on the private wage share.

Finally, I analyse the relevance of the results from the policy viewpoint. Considering that the different spending programmes have a significant effect on private wage share, to what extent do they contribute to the change in private wage share over the period and in relation to globalisation? To address this question, I construct a measure of each factor’s contribution by multiplying its effect size by its actual change over the period of interest. The cumulative change is computed as the yearly change of the variable time the number of years. The effect estimates are taken from the AB model in specification III (Table 2). The expenditures on government wages substantially contributed to curbing the fall of the private wage share by 1.6 points, while the expenditures on capital formation by 0.6 points. Conversely, trade openness contributed to the fall of the private wage share by 1.2 points. However, while state policy did curb the falling trend of the private wage share through government wages, it also accelerated it by increasing the spending on outsourcing (−0.7). The overall contribution of spending policy is still positive and amounts to 1 point. Other areas of policy intervention did not contribute to a similar degree to the private wage share. Indeed, wage coordination settings, as well as the degree of employment protection, did not change significantly between 1985 and 2010 in our panel.

**Table 2.** Impact on private wage share of GDP (AMECO) of spending programmes, labour market institutions, globalisation and technological change: OECD Countries.

	I (1985–2010)			II (1985–2010)			III (1985–2010)			IV (1985–2007)		
	Prais–Winsten FE	Prais–Winsten FD	Arellano–Bond	Prais–Winsten FE	Prais–Winsten FD	Arellano–Bond	Prais–Winsten FE	Prais–Winsten FD	Arellano–Bond	Prais–Winsten FE	Prais–Winsten, FD	Arellano–Bond
Government wages	0.74*** (0.16)	1.39*** (0.22)	0.41*** (0.08)	0.68*** (0.16)	1.37*** (0.22)	0.41*** (0.08)	0.60** (0.18)	1.08*** (0.21)	0.37*** (0.08)	0.53** (0.19)	1.04*** (0.25)	0.39*** (0.09)
Government outsourcing	−0.42** (0.15)	−0.62* (0.27)	−0.15~ (0.09)	−0.37* (0.14)	−0.62* (0.26)	−0.15~ (0.09)	−0.41** (0.15)	−0.48~ (0.28)	−0.19** (0.09)	−0.85*** (0.21)	−0.98** (0.36)	−0.52*** (0.11)
Government capital formation	0.34* (0.17)	0.27 (0.22)	0.05 (0.12)	0.31~ (0.16)	0.26 (0.21)	0.05 (0.12)	0.42** (0.16)	0.36~ (0.21)	0.12 (0.12)	0.40~ (0.24)	0.55* (0.28)	0.12 (0.16)
Unemployment benefits	−0.48 (0.29)	−0.50 (0.51)	−0.26 (0.20)	−0.42 (0.30)	−0.43 (0.52)	−0.26 (0.20)	−0.40 (0.33)	−0.62 (0.48)	−0.27 (0.19)	−0.92* (0.35)	−0.76 (0.50)	−0.44* (0.22)
Unemployment rate	−0.37*** (0.08)	−0.31** (0.09)	−0.24*** (0.05)	−0.33*** (0.08)	−0.30** (0.09)	−0.24*** (0.05)	−0.30*** (0.08)	−0.26** (0.08)	−0.22*** (0.05)	−0.10 (0.09)	−0.18~ (0.09)	−0.10~ (0.05)
Ln (GDP capita)	−60.90* (33.50)	−58.54 (65.47)	−18.90 (20.65)	−53.21 (34.28)	−59.34 (65.41)	−18.90 (20.77)	−42.32 (34.95)	−103.31 (62.77)	−11.24 (20.57)	−179.12** (62.68)	−239.10* (101.96)	−4.30 (29.04)
Ln (GDP capita)²	2.15 (1.65)	1.65 (3.17)	0.70 (1.01)	1.81 (1.68)	1.70 (3.16)	0.70 (1.02)	1.46 (1.72)	3.98 (3.03)	0.41 (1.01)	8.71** (3.12)	11.12* (5.00)	0.37 (1.42)
Industrial employment	−0.24* (0.10)	0.03 (0.13)	−0.15* (0.06)	−0.23* (0.10)	0.03 (0.13)	−0.16* (0.06)	−0.16 (0.10)	0.08 (0.12)	−0.14* (0.06)	−0.05 (0.09)	0.10 (0.11)	−0.11~ (0.06)
Wage coordination				0.10 (0.09)	0.06 (0.09)	0.03 (0.10)	0.07 (0.10)	0.03 (0.10)	0.01 (0.10)	0.26* (0.12)	0.17 (0.11)	0.06 (0.10)
Employment protection				2.83*** (0.66)	1.11 (0.90)	−0.01 (0.54)	2.35** (0.71)	1.01 (0.83)	−0.17 (0.54)	0.72 (0.79)	0.28 (0.97)	−0.39 (0.51)
Trade openness							−0.06*** (0.01)	−0.08*** (0.02)	−0.02*** (0.01)	−0.11*** (0.01)	−0.10*** (0.02)	−0.06*** (0.01)
ICT										3.81 (2.61)	0.41 (1.48)	0.82 (0.55)
ICT²										−0.30* (0.16)	4.20 (2.91)	−0.02 (0.08)
Private wage share (t-1)			0.77*** (0.03)			0.77*** (0.03)			0.75*** (0.03)			0.70*** (0.03)
Country-fixed effects	Yes	No	No	Yes	No	No	Yes	No	Yes	Yes	No	Yes
Year fixed effects	Yes	Yes	Yes	Yes	Yes	Yes	Yes	Yes	Yes	Yes	Yes	Yes
PCSEs	Yes	Yes	No	Yes	Yes	No	Yes	Yes	No	Yes	Yes	No
*R*^2^ (adjusted)	0.61	0.49	0.89	0.64	0.49	0.89	0.66	0.55	0.89	0.82	0.45	0.94
*N*	379	358	358	379	358	358	379	358	358	268	253	253
Countries	19	19	19	19	19	19	19	19	19	14	14	14

AMECO: Annual Macro-Economic Database; FE: country fixed effects; FD: first difference transformation; Ln: variable is log transformed; ICT: information and computer services; PCSEs: panel-corrected standard errors; GDP: gross domestic product.

Prais–Winsten: feasible generalised least squares Prais–Winsten estimator; Arellano–Bond: Arellano–Bond estimator (Arellano and Bond, 1991).

~0.1; *0.05; **0.01; ***0.001.

## Conclusion

This study analysed the impact of spending policy on the decline of the private wage share of income, distinguishing between the production-related expenditures and unemployment benefits. The proponents of power relations theory (Esping-Andersen, 1985; Hicks, 1999; Korpi, 2006) did not pay much attention to the consequences of the different spending programmes for the bargaining relations between labour and capital. We know that welfare state systems counteract the capitalist tendency to treat labour as a commodity (Esping-Andersen, 1990), yet an in-depth analysis of the consequences of spending programmes on the bargaining relations between capitalists and labour is lacking. Building on this tradition of research, this article distinguishes between types of expenditures that enhance the bargaining position of labour by creating better alternatives to low-paid jobs in the private sector and unemployment – that is, unemployment benefits and public sector employment – and labour-saving and pro-business types of expenditures – that is, outsourcing to private firms the provision of public services.

This article is in line with the focus on the balance of power between labour and capital of recent sociological research on wages (Kollmeyer, 2017; Kristal, 2013a; Sakamoto and Kim, 2014). Yet it argues that in addition to traditionally researched factors such as union activity, wage coordination settings, employment protection legislation and left parties, the bargaining power of labour is affected by spending policy. According to this view, state policy affects labour and capital’s agency not only through its effect on labour market institutions but also indirectly through its role in creating jobs and involving private firms in the provision of public services.

The results from panel regression strongly confirm that expenditures on government wages contributed substantially to curb the falling trend of the private wage share and that this contribution was independent of the effects of labour market institutions, globalisation and technological change. The results also show that the expenditures on capital formation had a positive effect on the private wage share of income, although the contribution to the change in private wage share was smaller than that of expenditures on government wages. The expenditures on outsourcing to private firms, by contrast, accelerated declining private wage share. The results do not support the hypothesis regarding the positive relationship between unemployment benefits and the private wage share. The results show instead an insignificant and negative relationship between unemployment benefits and the private wage share, which indicates that unemployment benefit trends reflected mostly changes in unemployment rates.

A limitation of this study is that it cannot capture the effect of institutional characteristics that are time-invariant. The within-country modelling approach, while providing more consistent and unbiased estimates of the average effectiveness for the 19 countries, cannot account for the effect of more stable characteristics such as the welfare state regime. The implication of the notion of welfare state for the presented study is that the variation in expenditures on public sector employment versus outsourcing could reflect country differences in the welfare state regime. We know that the conservative type tends to make benefits dependent on previous contributions and the liberal type tends to limit its social policies to the alleviation of poverty, while the social democratic type is committed to equalising both opportunities and outcomes across social strata (Esping-Andersen, 1990). These welfare state differences shape the allocation of public resources between the spending programmes and account for at least some of the between-country differences in the distribution between labour and capital. The use of a within-country approach precludes a comparison between welfare state regimes but does not detract from the validity of the results, with the caveat that the results refer to the average effectiveness for the 19 countries. This approach follows existing analyses of income inequality both within sociology and economics (Kristal, 2010; Lee et al., 2011; Stockhammer, 2009, 2015). In relation to the generalisability of the results, it should also be mentioned that our sample of 19 developed nations does not allow inferences to other developed countries.

Notwithstanding those limitations, the results suggest several important implications. First, the increased involvement of private firms in the provision of public services is an important component of the spending policies in advanced economies over the last three of four decades. It is not always recognised that attempts to reduce the budget deficit are accompanied by comparatively larger guaranteed flows of income to private firms, through the outsourcing of state functions.

Second, these results suggest that the treatment of government spending as simply social spending or public sector employment level can hide important differences between the distributional impacts of the different spending programmes. Transfers to households, government wages, government spending on outsourcing and capital formation should be treated as distinct types of spending in the analysis of income inequality.

Third, the consequences of spending policy for economic inequality are broader than the notion of decommodification suggests. Apart from social transfers, the expenditures on public sector employment also exert an enhancing effect on the social position of labour by providing options of good job opportunities outside the private sector. The labour-enhancing effect of the public sector employment was strong, even after controlling for the wage coordination settings, union activity and employment protection legislation, which are traditionally used to capture the bargaining power of labour in the economic sphere. The diverse and strong relevance of different spending programmes for distributional issues suggests that the flow of funds from the government to the actors in the private sector is an integral part of the political economy of income distribution. This implies that the effect of state policy on labour and capital’s agency is stronger than an exclusive focus on labour market institutions suggests as it includes the indirect effect exerted via spending policy.

Therefore, if the policy goal is to enhance the bargaining position of labour and increase its share of income, spending policy should prioritise the expenditures on the public sector employment.^10^ This implication provides support to the Keynesian argument in favour of investment in public sector employment rather than any kind of expansionary fiscal policy (Tcherneva, 2012). Reversing austerity policies by increasing spending regardless of the types of programmes may not be enough to ameliorate income inequality or could even enlarge the economic divide between capitalists and workers. Expenditures on public sector employment should grow faster than expenditures on outsourcing intermediate inputs to private firms. This implication is particularly relevant in light of the recent cuts to some sectors of public employment in several OECD countries while increasing the involvement of the private sector in delivering services historically dominated by the government.

## Appendix 1

**Table 3.** Descriptive statistics of 19 OECD countries, 1985–2010.

	Mean	Standard deviation	Minimum	Maximum
Private wage share	51.52	6.07	35.50	65.10
Government wages	9.53	2.46	4.32	18.22
Government outsourcing	5.14	1.90	1.80	12.29
Government capital formation	2.44	1.00	0.64	6.87
Unemployment benefits	1.19	0.76	0.20	3.49
Unemployment rate	7.20	3.05	2.00	20.03
Ln (GDP capita)	10.25	0.23	9.64	10.88
Ln (GDP capita)²	105.11	4.64	92.94	118.37
Industrial employment	17.95	3.10	11.50	27.38
Wage coordination	3.10	1.29	1.00	5.00
Employment protection	2.09	0.91	0.26	4.58
Trade openness	61.63	33.24	9.88	176.92
ICT	1.60	0.95	0.31	5.69

OECD: Organisation for Economic Co-operation and Development; Ln: variable is log transformed; ICT: information and computer services; GDP: gross domestic product.

## Notes

1. There are other types of social transfers, such as old-age pensions, sickness benefits and child benefits, but the main source of cash transfer that can lower the economic cost of unemployment are the unemployment benefits. For simplicity, I will only consider the unemployment benefits.

2. Data on wages by skill categories and by sectors were obtained from the European Union (EU) KLEMS datasets for 12 countries over the period 1985–2004. The countries are Austria, Belgium, Denmark, Finland, France, Germany, Italy, Japan, the Netherlands, Spain, Sweden and the United Kingdom. Skill categories are constructed using individual country-specific census data on the basis of workers’ educational qualifications. The high-skilled group includes workers with a college/university degree and above. The intermediate skill group includes workers with secondary-level qualifications that typically start after the 8th school year. The low-skilled group includes workers with no formal qualifications and primary education only.

3. The types of outsourced services provided by the government contractors include cleaning services, provision of equipment (which is not part of capital), clothing and uniforms, repair and maintenance services, meals for government workers, transportation/accommodation for travellers, research consultants and other overhead costs. Cleaning services is an example of a function which was historically dominated by government employees and which now, in several countries, is outsourced to private firms, which are likely to offer lower salaries than comparable low-skilled jobs in the government sector.

4. The choice of the period 1985–2010 is dictated by data availability. Spending programmes at this level of breakdown are not available for years earlier than 1985 for most countries, and the Comparative Welfare State dataset – which is the source of many variables – does not go beyond 2010 for some of the variables.

5. Expenditures data were derived from the Organisation for Economic Co-operation and Development (OECD) National Accounts Statistics, which are based on the System of National Accounts (SNA), a set of internationally agreed definitions for national accounting.

6. In this panel, the use of union coverage (employees covered by wage bargaining agreements as a percentage of all employees) as a measure of the influence of union is precluded by the reduced number of countries where this variable is available for a sufficient number of years (Comparative Welfare State dataset).

7. This implies that there is an association between the panel-level error and the lagged dependent variable.

8. The estimator deals with the autocorrelation not only by using the lagged dependent variable as a predictor but also by taking into consideration that the errors follow a first-order (*t−*1) moving average pattern. Assuming that the errors might be persistent between *t* and *t−*1 implies that only lags three or higher are valid instruments for the difference equation.

9. First differences remove factors that do not change over time, such as country dummies, and therefore do not allow country fixed effects.

10. As any increase in government spending, an employment-led spending policy is to be valued against other goals and constraints, including inflationary pressures and budget goals.
